# Prognostic and Clinic Pathological Value of Cx43 Expression in Glioma: A Meta-Analysis

**DOI:** 10.3389/fonc.2019.01209

**Published:** 2019-11-12

**Authors:** Chao Zhang, Cheng-fen Liu, An-bin Chen, Zhong Yao, Wei-guo Li, Shu-jun Xu, Xiang-yu Ma

**Affiliations:** ^1^Department of Neurosurgery, Qilu Hospital, Shandong University, Jinan, China; ^2^Brain Science Research Institute, Shandong University, Jinan, China

**Keywords:** glioma, Cx43, gap junctional intercellular communication, glioma survival time, meta-analysis

## Abstract

Gap junctional intercellular communication (GJIC) composed of connexin proteins is considered vital to cancer onset and progression since 50 years ago based on Lowenstein and Kano's works, however altered expression of connexins is still a lesser known “hallmark” of cancer. Although many studies support the hypothesis that connexins are tumor suppressors, recent evidence indicates that, in some tumor types including glioma, they may play contradictory role in some specific stages of tumor progression. We thus conduct a meta-analysis to evaluate the prognostic role of Cx43 in glioma for the unanswered questions that whether Cx43 is a beneficial or insalubrity factor for glioma. Eight studies with 1,706 patients were included for meta-analysis. The results showed that Cx43 expression was a clearly negative factor with tumor grades (*I*^2^ = 34%, *P* < 0.001) and beneficial for OS (*n* = 3, HR 2.62, 95%CI 1.47–4.68; *P* = 0.001). Subgroup analysis also found that Cx43 had different expression in Asian young patients vs. other groups. In conclusion, this article summarize the prognostic value of Cx43 and offer a clinical evidence for the notion that Cx43 is generally a tumor suppressor and beneficial for the patients' survival time.

## Introduction

Brain tumors account for 1.9% of all new cancer cases and 2.3% of cancer related deaths globally. Among them, glioblastoma (GBM) is the most common primary malignant brain tumor (more than 45% of all malignant brain tumors) (1). However, the 5-year survival individuals with traditional treatment is still <5% since past decades, ranking it the 6th most lethal of all types among tumors (2). Standard procedure of care for newly diagnosed GBM includes maximum surgery resection, followed by ionizing radiation and chemotherapy with temozolomide (TMZ). Evidences showed that surgery resection is still the most effective way to cure GBM and excision extension is closely tied to patients' overall survival, so the importance of early-diagnosing GBM and exact-safe resection appeal to more and more focus (3). Therefore, it is necessary to identify molecular markers to help to distinguish surgical range and give a predicted clinical outcomes of high risk patients.

Cellular communication is vital in numerous processes critical for tumor biological homeostasis, for cell survival, proliferation, differentiation, and invasion. And in turn, it facilitated disease through a passive intercellular transmit of small molecules, second messengers, ions, microRNAs, and electrical signals (4). The structure of Connexins are tetra-span integral plasma membrane, consisting of two extracellular loops and one intracellular loop along with cytoplasmic amino (NH_2_) and carboxyl-terminal (CT) tail domains. Up to now, numbers of Cx molecules have been explored in the CNS: Cx30, Cx32, Cx36, and Cx43 in neurons; Cx30, Cx40, Cx43, and Cx45 in astrocytes; Cx32, Cx36, and Cx43 in microglia; and Cx26, Cx32, Cx29, Cx36, and Cx47 in oligodendrocyte (5, 6).

Although the big family consisting of Connexins, Cx43 is one of the most important subtypes which highly expressed in GBM tissues in some patients and appeared to be a marker distinguishing glioma from other types of brain cancers such as oligodendrocytic (7, 8). Cx43 has also been reported to possess the tumor-suppressor-like activities, to cause cancer cell dedifferentiation (mesenchymal to epithelial transition), and to inhibit metastasis thus affecting the tumor progression (6). Recently, few cases reported that Cx43 expression was inversely correlated to tumor grade as a result Cx43 was considered to “normalize” the phenotype of rat and human glioma cells (4, 9, 10). However, these articles failed to find any association between Cx43 and OS in glioma in their study and no meta-analysis reported that. In order to give a convincing evidence for this issue, we thus gathered the most recent studies and performed a meta-analysis to pool the results from eligible studies to present a quantitative calculation.

## Methods

### Literature Search Strategy

A systematic review and meta-analysis was conducted under the recommends of PRISMA guidelines. Data extraction was systematically performed in PubMed, Web of Science and EMBASE/Medlin, with different combinations of the following key words [“Glioma,” “brain tumor” OR “glioblastoma”] AND [“Connexin43,” “gap junction channels 43” OR “Cx43”] and no language restrictions. Terms [“Glioma,” “brain tumor” OR “glioblastoma”] AND [“Connexin,” “gap junction channels” OR “Cx”] were also searched and after eliminating papers focusing on other Cx proteins, the included results were same as the combination of glioma and Cx43. This search was finally renewed till May 1, 2019. References consists of studies, clinical cases and review articles were also included to find additional patients data.

### Inclusion Criteria

Our purpose of this study was to identify whether Cx43 expression decreased the risk of glioma. Therefore, the criteria for enrolled study was as follows: (1) study should concerned the relationship between Cx43 and clinical outcomes among patients with gliomas. (2) Gliomas should be diagnosed by standard criteria, histopathologic analysis. (3) Cx43 expression was examined in glioma tissue obtained from glioma patients not experimental cells. (4) Cx43 expression was examined by immunohistochemistry (IHC) methods, PCR or tissue microarray. (5) The data should provide detailed information to calculate overall survival (OS) or disease free survival (DFS). Studies that could not meet any one of the above inclusion criteria were excluded. Studies which did not meet the above requirements were excluded and animal studies, letters were also precluded.

### Data Extraction

Two authors independently evaluated all studies and selected eligible trials, and any discrepancies between the authors were resolved by discussion and consensus. Among the studies, data was extracted included following information: country of the population enrolled, first author's name, number of included patients, and year of publication, sample size, patients' age, histological grade, diagnosed technique, and positive standard, antibody used, overall survival rates and survival outcomes. Kaplan-Meier (K-M) curves of these studies were analyzed by Engauge Digitizer (http://sourceforge.net/projects/digitizer/) which was utilized to calculate the OR and DFS. When univariate analysis and multivariate analysis were all provided in studies, we prefer the former results to be analyzed in the study. If the extract information could not be retained directly from included studies, we would send an email to the correspond author to get the original relevant data, otherwise the item would be marked as “Not Documented (ND).”

### Statistical Analysis

The Revman (version 5.3) was used to conduct all the calculations during the whole process of meta-analysis. Odds risks (OR) with 95% confidence intervals (CIs) were calculated and conducted to evaluate the correlation between Cx43 and clinical pathological outcomes in forest plots. *I*^2^ test and Q test were used to estimate heterogeneity in these studies. If the *I*^2^ test (>50%) or the Q test (*P* < 0.05) were abnormal, which indicated a significant heterogeneity between the selected studies, random-effect model would be introduced to assess the results, and otherwise the fixed-effect model would be used. Sub-group analyses based on research techniques (IHC or PCR), ethnicity and sample size were conducted. Sensitivity analysis was conducted to evaluate heterogeneity and stability of enrolled data. Potential publication bias were assessed by the funnel plots and Egger's tests. Also the effect of Cx43 diagnostic sensitivity and specificity were also presented by forest plot and SROC curve.

## Results

### Study Selection and Characteristics

**Figure 1:**
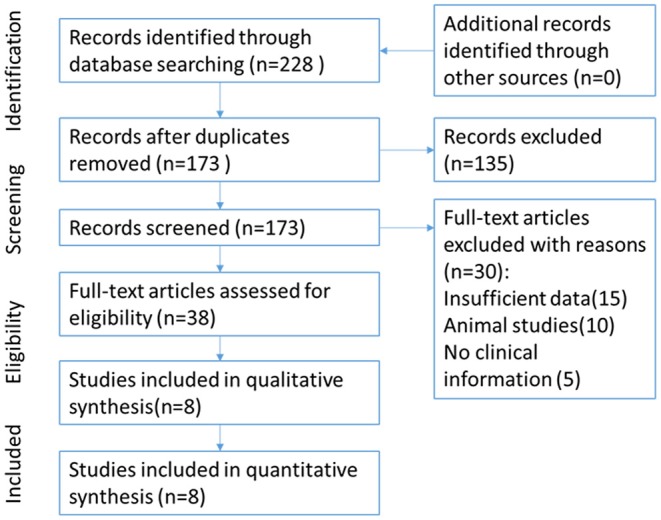
Flow chart for selection of studies.

The flow of study selection has been presented in Figure 1. Based on an extensive combination of keywords search and screened a total of 173 papers by article title as well as abstract, we picked up 8 (9, 11–17) published studies that fulfilled all inclusion criteria which required intact data, strict experiment design and minor publication bias in the present meta-analysis. The enrolled studies were well-controlled and accorded with selection criterions. Based on the expression level of Cx43, enrolled patients of all the studies were divided into different subgroups: the high Cx43 level patients confirming to every positive standard in papers were classified into the Cx43 High subgroup, and patients with low Cx43 levels were attributed into the Cx43 Low subgroup. Overall, 8 studies constituting 790 Cx43 High patients and 916 Cx43 Low patients were evaluated with tumor grades, ethnicity, research technique and overall survival (OS).

### Study Characteristics and Quality Assessment

The enrolled studies and clinical characteristics of included articles are presented in Table 1. Eight studies were conducted within western countries, and two within Asia. Five studies including more than 100 patients while the other three studies had relatively smaller patient's numbers. Two studies examined Cx43 expression by RT-PCR and six studies used IHC methods. Three articles evaluated cancer survival and recurrence. The publication time of all papers ranged from 2003 to 2016. The number size of enrolled group ranged from 32 to 572, and the positive rates of Cx43 expression varied from 55.6 to 89.2%. To examine the quality of included studies, Newcastle-Ottawa Quality Assessment scores (NOS) were introduced and the data ranged from six to nine (detail listed in Table 2), which manifested that the quality of enrolled studies was high. Exacted clinical data could be browsed in Tables 1, 2.

**Table 1:** Characteristics of included studies into meta-analysis.

**Author**	**Ethnicity**	**Year**	**Sample size**	**Age (yr)**	**Histology**	**Research techniques**	**Ab used**	**Positive standard**
Peiyu Pu	China	2003	52	ND	Normal 8 Grade I 4 (pilocytic astrocytoma) Grade II 12 (protoplasmic and fibrillary astrocytoma) Grade III 14 (anaplastic astrocytoma) Grade IV 14 (glioblastoma multiforme)	IHC	Santa Cruz	No positive cells (0), positive cells <25% (1), between 25 and 50% (2), between 50 and 75% (3), >75% (4)
Rosario Caltabiano	Italy	2010	32	43.9 (2–80)	Grade I 7 Grade II 5 Grade III 7 Grade IV 13	IHC RT-PCR	Zymed	Negative (–) = absence of labeling, (+) = positivity <50% of the neoplastic glial cells, (++) = positivity from 50 to 100% of the neoplastic glial cells.
Paul R. Gielen	America	2013	208	45.5 (9–70)	Normal 8 Grade I 13 Grade II 42 Grade III 112 Grade IV 33	IHC RT-PCR	Sigma, C6219	No positive cells (0), positive cells <25% (1), between 25 and 50% (2), between 50 and 75% (3), >75% (4)
Joanna Reszeć	Poland	2014	131	65.9	Grade II 26 (diffuse astrocytoma anaplasic) Grade III 44 (astrocytomas) Grade IV 61 (glioblastoma)	IHC	Santa Cruz, C-20	≤ 10% positive cells (–), 11–50% (+), ≥51% (++)
Susan F. Murphy	America	2015	520	ND	Normal 62 Grade I 95 Grade II 214 Grade III 80 Grade IV 121	IHC RT-PCR	Sigma	≤ 10% positive cells (–), 11–50% (+), ≥51% (++)
WC Sin	Canada	2015	474	42.9 (2–72)	Normal 52 Grade I 86 Grade II 205 Grade III 71 Grade IV 112	IHC	Sigma,C6219	Negative (–) = absence of labeling, (+) = positivity <50% of the neoplastic glial cells, (++) = positivity from 50 to 100% of the neoplastic glial cells.
Xin-Yun Ye	China	2015	80	ND	Grade I 20 (pilocytic astrocytoma) Grade II 20 (protoplasmic and fibrillary astrocytoma) Grade III 20 (anaplastic astrocytoma) Grade IV 20 (glioblastoma multiforme)	IHC	Santa Cruz	No positive cells(0), positive cells <25% (1), between 25 and 50%(2), between 50 and 75% (3), >75% (4)
Sophie Crespin	France	2016	85	51.9 (16–78)	Grade II 19 Grade III 12 Grade IV 22	IHC	Transduction laboratories, USA	No positive cells (0), positive cells <25% (1), between 25 and 50% (2), between 50 and 75% (3), >75% (4)

*ND, no details; IHC, immunohistochemistry; PCR, polymerase chain reaction*.

**Table 2:** Newcastle-Ottawa Quality Assessment Scale of included studies.

	**Selection**				**Comparability**	**Outcome**			
**Study**	**Represen-tativeness of exposed cohort 1**	**Selection of non-exposed group 2**	**Ascertartainment of expose 3**	**Outcome of interest 4**	**Comparability of cohorts 5**	**Assessment of outcome 6**	**Length of follow-up 7**	**Adequency of follow-up 8**	**Score**
Peiyu Pu	1	1	1	1	1	1	0	0	6
Rosario Caltabiano	1	1	1	1	1	1	0	0	6
Paul R. Gielen	1	1	1	1	2	0	1	1	8
Joanna Reszeć	1	1	1	1	1	1	0	0	6
Susan F. Murphy	1	1	1	1	2	0	1	1	8
W. C. Sin	1	1	1	1	2	0	1	1	8
Xin-Yun Ye	1	1	1	1	1	1	0	0	6
Sophie crespin	1	1	1	1	1	1	0	0	6

### Association Between Cx43 in Glioma and 3-Year Overall Survival

**Figure 2:**
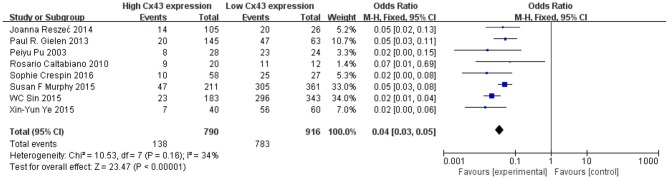
Forest plot of studies evaluating the relationship between Cx43 expression and tumor grades.

**Figure 3:**
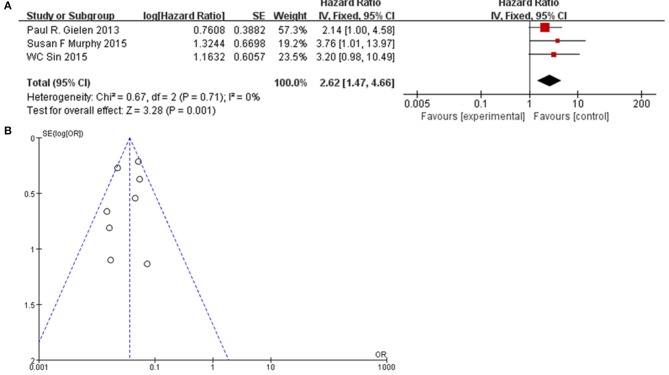
**(A)** Forest plot of the association between Cx43 and OS in glioma patients. **(B)** Funnel plot for publication bias test of Cx43 related studies.

Methods described previously were introduced to evaluate the results, the association between Cx43 and tumor grades were estimated by forest plots. Cx43 showed clearly negative with tumor grades in glioma patients (*I*^2^ = 34%, *P* < 0.001) (Figure 2). Three studies enrolled 1,202 patients were examined for the relationship between Cx43 and 3-year OS. And data (Figure 3A) showed that less Cx43 level was related with poor prognosis of glioma patients (HR 2.62, 95%CI 1.47–4.68; *P* = 0.001). Otherwise, this meta-analysis indicated that Cx43 level was highly related with a higher OS rates. The *P* value and *I*^2^ of this results showed they were eligible. There was no notable heterogeneities among these studies likely existed [Heterogeneity: Chi^2^ = 10.53, *df* = 7 (*P* = 0.16); *I*^2^ = 34%] (Figure 3B), so the resulting was reliable and then random-effect model was introduced to assess the possible publication bias of clinic pathological features.

### Association Between Cx43 and Clinic Pathological Features

**Figure 4:**
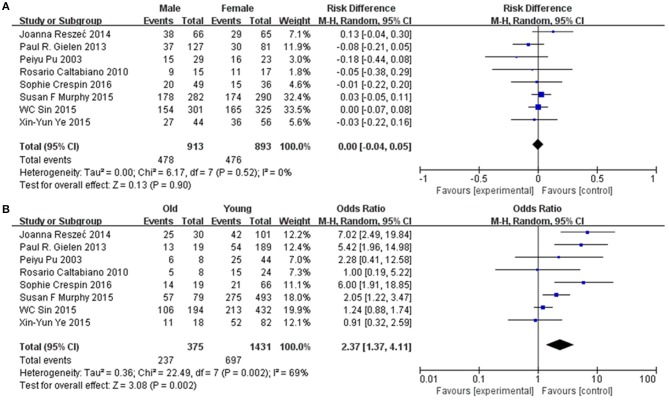
Association between Cx43 and clinical pathological features in glioma patients. **(A)** Gender. **(B)** Age.

To further confirm the potential value of Cx43 in clinical practice, the relationship between Cx43 and patients' clinic pathological characters including tumor grades, gender and average age were explored precisely. As seen in Table 3 and Figure 4, forest plots of 8 eligible studies showed the downregulated Cx43 was associated with tumor grades (*P* < 0.001). No difference was found between Cx43 expression in gender group (*P* = 0.86) (Figure 4A), but in age group, it effected the positive rate of Cx43 (*P* = 0.002). The difference may come from the different morbidity in different age and also the criteria for young (<60) and old (>60) do have influence on the bias (Heterogeneity: Chi^2^ = 22.49, *P* = 0.002; *I*^2^ = 69%) (Figure 4B).

**Table 3:** Data of subgroup for analyze of the gender and age effects.

	**Male**		**Female**		**Old**		**Young**	
	**Positive**	**Total**	**Positive**	**Total**	**Positive**	**Total**	**Positive**	**Total**
Reszec et al. (13)	38	66	29	65	25	30	42	101
Gielen et al. (12)	37	127	30	81	13	19	54	189
Pu et al. (11)	15	29	16	23	6	8	25	44
Caltabiano et al. (9)	9	15	11	17	5	8	15	24
Crespin et al. (17)	20	49	15	36	14	19	21	66
Murphy et al. (14)	178	282	174	290	57	79	275	493
Sin et al. (15)	154	301	165	325	106	194	213	432
Ye et al. (16)	27	44	36	56	11	18	52	82

### Sensitivity and Subgroup Analyses

**Figure 5:**
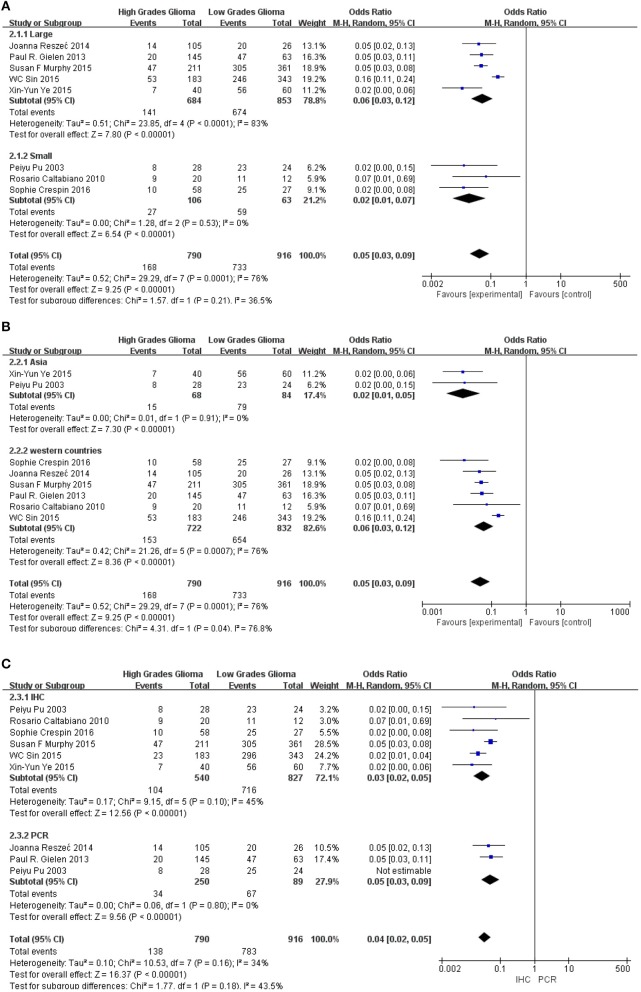
Subgroup analysis of Cx43 with ethnicity, sample size and research technique: **(A)** sample size; **(B)** region; **(C)** research technique.

Subgroup analysis was performed to explore the potential sources of heterogeneity, which were divided by research techniques, ethnicity and sample sizes. Table 4 and Figure 5 present the results of subgroup analyzed elucidate the correlations between Cx43 level and tumor grades. These results also indicated that low Cx43 expression was related to a significantly poorer results as compared to high expression. While sample size (*P* = 0.21) and research technique (*P* = 0.20) (IHC vs. PCR) did not obviously effect the prognosis rate of Cx43, but there might be a difference between Asian group and Western country group (Chi^2^ = 4.31, *P* = 0.04, *I*^2^ = 76.8%) (Figure 5B).

**Table 4:** Subgroup analysis of region, sample size, and research technique.

**Factors**	**Studies**	**Patients**	**Effect model**	**Test for subgroup differences:**	**Heterogeneity:**
**Region**					
Asian countries	2	152	Odds Ratio (M-H, Randomed, 95% CI)	Chi^2^ = 4.31, *df* = 1 (*P* = 0.04), *I*^2^ = 76.8%	Chi^2^ = 29.29, *df* = 7 (*P* = 0.0001); *I*^2^ = 76%
Non-Asian countries	6	1,554	Odds Ratio (M-H, Randomed, 95% CI)		
**Sample size**					
>100	5	1,537	Odds Ratio (M-H, Randomed, 95% CI)	Chi^2^ = 1.57, *df* = 1 (*P* = 0.21), *I*^2^ = 36.5%	Chi^2^ = 29.29, *df* = 7 (*P* = 0.0001); *I*^2^ = 76%
<100	3	169	Odds Ratio (M-H, Randomed, 95% CI)		
**Research technique**					
IHC	6	1,367	Odds Ratio (M-H, Randomed, 95% CI)	Test for subgroup differences: Chi^2^ = 1.77, *df* = 1 (*P* = 0.18), *I*^2^ = 43.5%	Chi^2^ = 10.53, *df* = 7 (*P* = 0.16); *I*^2^ = 34%
RT-PCR	2	339	Odds Ratio (M-H, Randomed, 95% CI)		

### Publication Bias

Potential publication bias was evaluated by using Begg's funnel plot and Egger's test. The results were presented in Figure 5. The results indicated that there was no significant publication bias for all studies, and no evidence of significant publication bias was found in this paper.

**Figure 6:**
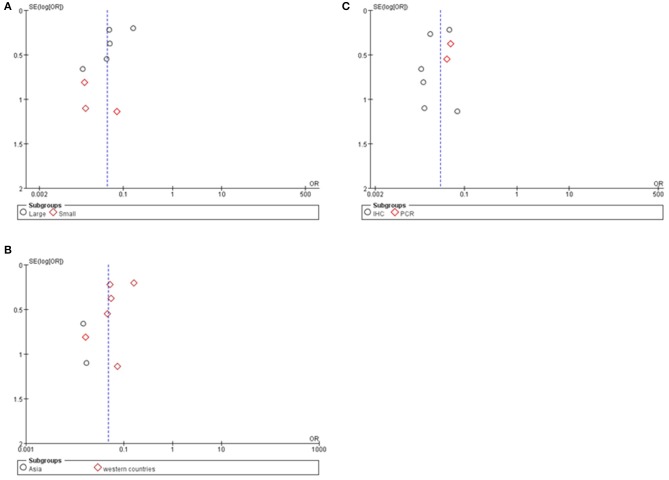
Funnel plot for publication bias test of Cx43 related studies. **(A)** Sample size. **(B)** Region. **(C)** Research technique.

As seen in Figure 6 and Table 4, group size didn't I effect the correlation between Cx43 level and tumor grades (*Test for subgroup differences: Chi*^2^ = *1.57, P* = *0.21, I*^2^ = *36.5%*). However, the heterogeneity of ethnicity existed in these subgroups (*Heterogeneity: Chi*^2^ = *29.29, P* = *0.0001; I*^2^ = *76%*). Additionally, research technique didn't influence the conclusion. And there were little heterogeneity among these subgroups (*IHC*: *I*^2^ = 45%, *P* = 0.10; *RT-PCR*: *I2* = 0%, *P* = 0.80). While in both subgroups divided by ethnicity, Cx43 expression was correlated to patients' ethnicity (*Test for subgroup differences: Chi*^2^ = *4.31, P* = *0.04, I*^2^ = *76.8%*). Heterogeneity in these subgroups was obvious, so further studies were still needed (*Heterogeneity: Chi*^2^ = *29.29, P* = *0.0001; I*^2^ = *76%*). With these results, the heterogeneity of tumor grades mainly resulted from the sample size and ethnicity.

### Cx43 Diagnostic Sensitivity and Specificity

**Figure 7:**
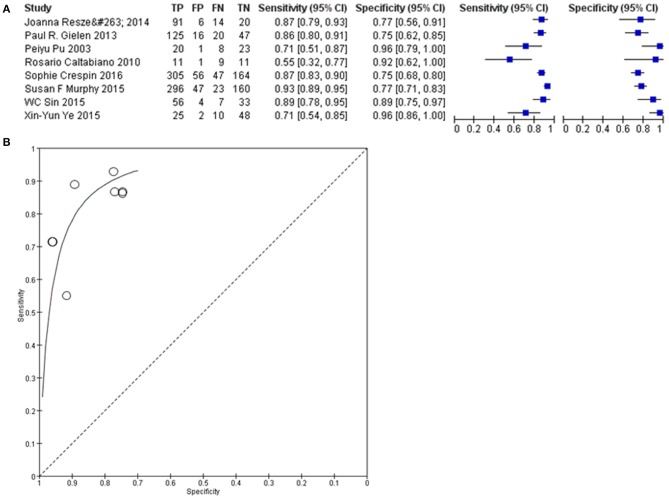
Diagnosis effect of Cx43 in glioma patients. **(A)** The sensitivity and specificity of Cx43 in glioma patients. **(B)** The diagnostic curve of Cx43 among glioma patients.

Sensitivity and specificity analyses were conducted to evaluate the diagnostic effect of Cx43 in glioma patients'. As seen in Figure 7, forest plot and SROC curve of 8 eligible studies showed the Cx43 is highly related to low grades glioma patients and shows good potential of sensitivity and specificity to define the tumor grades.

## Discussion

A huge number of studies have confirmed the relationship between cancer and connexins which was hypothesized more than 40 years ago (8, 18–20). However, the pivotal role of Cx43 in influencing tumor progression used to be overlooked because downregulation of Cx43-mediated intercellular communication is normally leading to increased malignancy in tumor cells (8, 19).As a result, Cx43 is usually considered as a tumor suppressor by its effect on reducing glioma proliferation (21, 22), anti-metastasis (23), and pro-apoptosis (24) and anti-inflammation (25). And our study is corresponded to these existing research that Cx43 expression is negatively associated with tumor grades.

In some studies, restoring Cx43 to glioma cells *in vitro* inhibits their tumorigenicity but this tumor suppressor effect could be glossed by its' promotion on invasion, adhesion and migration *in vivo* (1). Over-expression of Cx43 has been reported to enhance glioma migration in a channel-dependent manner, especially within the help of astrocytes (26, 27). Some experiments showed that Co-culture of glioma cells with astrocytes enhanced the invasiveness of the glioma cells and silencing Cx43 could extenuate this effect (25). Further study demonstrated this result and confirmed that junctions between glioma-glioma suppressed its invasiveness, while GJs of glioma–astrocyte and astrocyte–astrocyte promoted invasion (28). This seemed to be contrary to our findings that high Cx43 expression was beneficial to overall survive rate (OS). But it could be explained by the clinical experience that Cx43 usually highly expressed in low grades patients who could get gross-total resection (GTR) or even supra-total resection (SupTR) (29).

Due to nature of Cx43 that locates at the cell surface, Cx43 is associated with the brain endothelial barrier formation and the loss of Cx43 in glioma may lead to the infiltration of inflammation cells as well as more concentration of chemotherapy drugs (30). And further study of the correlationship between Cx43 and the popular glioma biomarkers is undergoing a proof study which would be published soon by our team. Besides Cx43 also the induction of inflammatory signaling, such as the treatment of anti-cancer agents and exposure to cytokines. One report demonstrated that Cx43 promoted glioma cell resistance to temozolomide by a channel-dependent (31), and another study find that Cx43 interacted Bax signal pathway by modulating mitochondrial apoptosis (32). Therefore, it will be beneficial to examine Cx43 expression in glioma before physician apply temozolomide or develop a drug to target Cx43. Cx43 peptidomimetics provide a potential method to selectively modulate the activity of connexin GJs as well as the numerous factors that correlated with tumorigenesis, recently study shown that inhibiting Cx43 restored TMZ sensitivity in TMZ-resistant/Cx43-high GBM cells including GSCs (33).

Besides, Cx43 is the most abundant Cx isoform in adult astrocytes especially in reactive astrocytes (34, 35). It was also demonstrated that reactive astrocytes such as M2 cell (abbreviation of pro-tumorigenic macrophage) is vital for innate immune systems in brain tumor microenvironment (36) and might also be responsible for immune therapy results. As a result, a high Cx43 expression is often seen in low grade gliomas at the early stage (15).

Cx43 channels are well known for the direct passage of small ions and metabolites such as Ca2+, ATP, glutamate, glucose and peptides through (37). However, emerging evidence has shown that Cx43 is also permeable to oligonucleotides as long as 24 nucleotides in length. Other results also demonstrated that glioma–astrocyte GJs were capable to transfer miRNAs (28). These findings adding to recent studies confirmed that cancer cells might “reprogram” the normal stromal cells by miRNAs through Cx43 junctions and normal cell might be hijacked by tumor cells to form a favorable environment for tumor as a result.

One unique character of Cx43 is the relationship with metabolism. Under glucose uptake situation, GJs allow the transition of glucose and other metabolic substrates throughout the network (38). Recently, studies find the inhibition of gap junctional communication or the decreasing of Cx43 expression resulted in an increase in the rate of glucose uptake (39) and aerobic glycolysis, which may be an explanation of poor prognosis of OS and high negative relevance ratio of Cx43 in high grade gliomas.

The role of Cx43 in tumor hemostasis is actually a very complex relationship. The mechanisms underlying these effects suggest a complex balance of variety proteins in the whole process (18). Our meta-analysis directly indicated that lower Cx43 as associated with poorer patient prognosis and thus revealed the potential value of Cx43 in diagnosis and prognosis of gliomas, however need more studies are still needed to elaborate the exact mechanism of Cx43 to bring it into clinical practice.

## Author Contributions

CZ wrote the main part of the manuscript including introduction results, methods and materials, and abstract. CL collected the related papers. AC and ZY assessed the quality of included papers. WL, SX, and XM revised the paper. All authors reviewed the manuscript.

### Conflict of Interest

The authors declare that the research was conducted in the absence of any commercial or financial relationships that could be construed as a potential conflict of interest.

## References

[1] Ferlay J Shin HR Bray F Forman D Mathers C Parkin DM. Estimates of worldwide burden of cancer in 2008: GLOBOCAN 2008. Int J Cancer. (2010) 127:2893–917. 10.1002/ijc.25516 21351269

[2] Ameratunga M Pavlakis N Wheeler H Grant R Simes J Khasraw M. Anti-angiogenic therapy for high-grade glioma. Cochrane Database Syst Rev. (2018) 11:Cd008218. 10.1002/14651858.CD008218.pub4 30480778 PMC6516839

[3] Sanai N Berger MS. Surgical oncology for gliomas: the state of the art. Nat Rev Clin Oncol. (2018) 15:112–25. 10.1038/nrclinonc.2017.171 29158591

[4] Grek CL Rhett JM Ghatnekar GS. Cardiac to cancer: connecting connexins to clinical opportunity. FEBS Lett. (2014) 588:1349–64. 10.1016/j.febslet.2014.02.047 24607540 PMC4031645

[5] Nakase T Naus CC. Gap junctions and neurological disorders of the central nervous system. Biochim Biophys Acta. (2004) 1662:149–58. 10.1016/j.bbamem.2004.01.009 15033585

[6] Giaume C Leybaert L Naus CC Saez JC. Connexin and pannexin hemichannels in brain glial cells: properties, pharmacology, and roles. Front Pharmacol. (2013) 4:88. 10.3389/fphar.2013.00088 23882216 PMC3713369

[7] Bai D. Structural analysis of key gap junction domains–Lessons from genome data and disease-linked mutants. Semin Cell Dev Biol. (2016) 50:74–82. 10.1016/j.semcdb.2015.11.015 26658099

[8] Naus CC Laird DW. Implications and challenges of connexin connections to cancer. Nat Rev Cancer. (2010) 10:435–41. 10.1038/nrc2841 20495577

[9] Caltabiano R Torrisi A Condorelli D Albanese V Lanzafame S. High levels of connexin 43 mRNA in high grade astrocytomas. Study of 32 cases with *in situ* hybridization. Acta Histochem. (2010) 112:529–35. 10.1016/j.acthis.2009.05.008 19604543

[10] Huang R Liu YG Lin Y Fan Y Boynton A Yang D. Enhanced apoptosis under low serum conditions in human glioblastoma cells by connexin 43 (Cx43). Mol Carcinog. (2001) 32:128–38. 10.1002/mc.1072 11746825

[11] Pu P Xia Z Yu S Huang Q. Altered expression of Cx43 in astrocytic tumors. Clin Neurol Neurosurg. (2004) 107:49–54. 10.1016/j.clineuro.2004.03.006 15567553

[12] Gielen PR Aftab Q Ma N Chen VC Hong X Lozinsky S. Connexin43 confers Temozolomide resistance in human glioma cells by modulating the mitochondrial apoptosis pathway. Neuropharmacology. (2013) 75:539–48. 10.1016/j.neuropharm.2013.05.002 23688923

[13] Reszec J Szkudlarek M Hermanowicz A Bernaczyk PS Mariak Z Chyczewski L. N-cadherin, beta-catenin and connexin 43 expression in astrocytic tumours of various grades. Histol Histopathol. (2015) 30:361–71. 10.14670/HH-30.361 25386667

[14] Murphy SF Varghese RT Lamouille S Guo S Pridham KJ Kanabur P. Connexin 43 inhibition sensitizes chemoresistant glioblastoma cells to temozolomide. Cancer Res. (2016) 76:139–49. 10.1158/0008-5472.CAN-15-1286 26542214 PMC5113032

[15] Sin WC Aftab Q Bechberger JF Leung JH Chen H Naus CC. Astrocytes promote glioma invasion via the gap junction protein connexin43. Oncogene. (2016) 35:1504–16. 10.1038/onc.2015.210 26165844

[16] Ye XY Jiang QH Hong T Zhang ZY Yang RJ Huang JQ. Altered expression of connexin43 and phosphorylation connexin43 in glioma tumors. Int J Clin Exp Pathol. (2015) 8:4296–306. 26191122 PMC4502994

[17] Crespin S Fromont G Wager M Levillain P Cronier L Monvoisin A. Expression of a gap junction protein, connexin43, in a large panel of human gliomas: new insights. Cancer Med. (2016) 5:1742–52. 10.1002/cam4.730 27306693 PMC4971902

[18] Tabernero A Gangoso E Jaraiz-Rodriguez M Medina JM. The role of connexin43-Src interaction in astrocytomas: a molecular puzzle. Neuroscience. (2016) 323:183–94. 10.1016/j.neuroscience.2015.02.029 25711938

[19] Mesnil M Crespin S Avanzo JL Zaidan-Dagli ML. Defective gap junctional intercellular communication in the carcinogenic process. Biochim Biophys Acta. (2005) 1719:125–45. 10.1016/j.bbamem.2005.11.004 16359943

[20] Aasen T Mesnil M Naus CC Lampe PD Laird DW. Gap junctions and cancer: communicating for 50 years. Nat Rev Cancer. (2017) 17:74. 10.1038/nrc.2016.142 28704356

[21] Saito T Sato H Virgona N Hagiwara H Kashiwagi K Suzuki K. Negative growth control of osteosarcoma cell by Bowman-Birk protease inhibitor from soybean; involvement of connexin 43. Cancer Lett. (2007) 253:249–57. 10.1016/j.canlet.2007.01.021 17343982

[22] Zhou JZ Riquelme MA Gu S Kar R Gao X Sun L. Osteocytic connexin hemichannels suppress breast cancer growth and bone metastasis. Oncogene. (2016) 35:5597–607. 10.1038/onc.2016.101 27041582 PMC5050050

[23] Kou Y Ji L Wang H Wang W Zheng H Zou J. Connexin 43 upregulation by dioscin inhibits melanoma progression via suppressing malignancy and inducing M1 polarization. Int J Cancer. (2017) 141:1690–703. 10.1002/ijc.30872 28677156

[24] Uzu M Sato H Yamada R Kashiba T Shibata Y Yamaura K. Effect of enhanced expression of connexin 43 on sunitinib-induced cytotoxicity in mesothelioma cells. J Pharmacol Sci. (2015) 128:17–26. 10.1016/j.jphs.2015.04.002 26003083

[25] Uzu M Sin WC Shimizu A Sato H. Conflicting roles of connexin43 in tumor invasion and growth in the central nervous system. Int J Mol Sci. (2018) 19:E1159. 10.3390/ijms19041159 29641478 PMC5979343

[26] Oliveira R Christov C Guillamo JS de Bouard S Palfi S Venance L. Contribution of gap junctional communication between tumor cells and astroglia to the invasion of the brain parenchyma by human glioblastomas. BMC Cell Biol. (2005) 6:7. 10.1186/1471-2121-6-7 15715906 PMC553963

[27] Lin JH Takano T Cotrina ML Arcuino G Kang J Liu S. Connexin 43 enhances the adhesivity and mediates the invasion of malignant glioma cells. J Neurosci. (2002) 22:4302–11. 10.1523/JNEUROSCI.22-11-04302.2002 12040035 PMC6758793

[28] Hong X Sin WC Harris AL Naus CC. Gap junctions modulate glioma invasion by direct transfer of microRNA. Oncotarget. (2015) 6:15566–77. 10.18632/oncotarget.3904 25978028 PMC4558171

[29] Roh TH Kang SG Moon JH Sung KS Park HH Kim SH. Survival benefit of lobectomy over gross-total resection without lobectomy in cases of glioblastoma in the noneloquent area: a retrospective study. J Neurosurg. (2019). 10.3171/2018.12.JNS182558. [Epub ahead of print]. 30835701

[30] Kaneko Y Tachikawa M Akaogi R Fujimoto K Ishibashi M Uchida Y. Contribution of pannexin 1 and connexin 43 hemichannels to extracellular calcium-dependent transport dynamics in human blood-brain barrier endothelial cells. J Pharmacol Exp Ther. (2015) 353:192–200. 10.1124/jpet.114.220210 25670633

[31] Kim SW Choi HJ Lee HJ He J Wu Q Langley RR. Role of the endothelin axis in astrocyte- and endothelial cell-mediated chemoprotection of cancer cells. Neuro Oncol. (2014) 16:1585–98. 10.1093/neuonc/nou128 25008093 PMC4232084

[32] Uzu M Sato H Shimizu A Shibata Y Ueno K Hisaka A. Connexin 43 enhances Bax activation via JNK activation in sunitinib-induced apoptosis in mesothelioma cells. J Pharmacol Sci. (2017) 134:101–7. 10.1016/j.jphs.2017.05.005 28602541

[33] Rhett JM Calder BW Fann SA Bainbridge H Gourdie RG Yost MJ. Mechanism of action of the anti-inflammatory connexin43 mimetic peptide JM2. Am J Physiol Cell Physiol. (2017) 313:C314–26. 10.1152/ajpcell.00229.2016 28701358 PMC5625091

[34] Szilvasy-Szabo A Varga E Beliczai Z Lechan RM Fekete C. Localization of connexin 43 gap junctions and hemichannels in tanycytes of adult mice. Brain Res. (2017) 1673:64–71. 10.1016/j.brainres.2017.08.010 28803831

[35] Kozoriz MG Bechberger JF Bechberger GR Suen MW Moreno AP Maass K. The connexin43 C-terminal region mediates neuroprotection during stroke. J Neuropathol Exp Neurol. (2010) 69:196–206. 10.1097/NEN.0b013e3181cd44df 20084014

[36] Kolar K Freitas-Andrade M Bechberger JF Krishnan H Goldberg GS Naus CC. Podoplanin: a marker for reactive gliosis in gliomas and brain injury. J Neuropathol Exp Neurol. (2015) 74:64–74. 10.1097/NEN.0000000000000150 25470350

[37] Simon AM Goodenough DA. Diverse functions of vertebrate gap junctions. Trends Cell Biol. (1998) 8:477–83. 10.1016/S0962-8924(98)01372-5 9861669

[38] Wolf A Agnihotri S Micallef J Mukherjee J Sabha N Cairns R. Hexokinase 2 is a key mediator of aerobic glycolysis and promotes tumor growth in human glioblastoma multiforme. J Exp Med. (2011) 208:313–26. 10.1084/jem.20101470 21242296 PMC3039857

[39] Herrero-Gonzalez S Valle-Casuso JC Sanchez-Alvarez R Giaume C Medina JM Tabernero A. Connexin43 is involved in the effect of endothelin-1 on astrocyte proliferation and glucose uptake. Glia. (2009) 57:222–33. 10.1002/glia.20748 18756537

